# Integrating anticipatory action in disease outbreak preparedness and response in the humanitarian sector

**DOI:** 10.1136/bmjgh-2024-017721

**Published:** 2025-07-23

**Authors:** Tilly Alcayna, Franziska Kellerhaus, Leo Tremblay, Chloe Fletcher, Rachel Goodermote, Mauricio Santos-Vega, Juan Chaves-Gonzalez, Meghan Bailey, V. Bhargavi Rao, Rachel Lowe

**Affiliations:** 1 Red Cross Red Crescent Climate Centre, The Hague, Netherlands; 2 Centre on Climate Change and Planetary Health, London School of Hygiene and Tropical Medicine, London, UK; 3 Centre for Mathematical Modelling of Infectious Diseases, London School of Hygiene and Tropical Medicine, London, UK; 4 Health in Humanitarian Crises Centre, London School of Hygiene and Tropical Medicine, London, UK; 5 German Red Cross, Berlin, Germany; 6 Médecins sans Frontières (MSF) Canada, Toronto, Ontario, Canada; 7 Barcelona Supercomputing Center (BSC), Barcelona, Spain; 8 Department of Medicine and Life Sciences, Universitat Pompeu Fabra, Barcelona, Spain; 9 International Federation of Red Cross and Red Crescent Societies, Geneva, Switzerland; 10 Universidad de los Andes, Bogota, Colombia; 11 United Nations Office for the Coordination of Humanitarian Affairs, New York, NY, USA; 12 Manson Unit, Médecins sans Frontières (MSF) UK Operational Centre Amsterdam, London, UK; 13 Catalan Institution for Research and Advanced Studies (ICREA), Barcelona, Spain

**Keywords:** Global Health, Environmental health, Prevention strategies, Infections, diseases, disorders, injuries, Cholera, Infectious Diseases, Climate

## Abstract

In the humanitarian sector, anticipatory action entails acting ahead of predicted hazardous events to prevent or mitigate potential impacts and needs. It leverages early warnings to bridge preparedness and response, with a core principle being the provision of ex-ante emergency funding for preagreed early actions. Traditionally applied to extreme climatic events, there is growing interest in integrating anticipatory action into disease outbreak preparedness and response. We present an analytical framework for trigger development for climate-sensitive infectious disease outbreaks based on a review of existing and emerging practices from the Red Cross Red Crescent Movement, United Nations agencies and Médecins Sans Frontières since 2014. We propose that, depending on data availability, there are four broad approaches for trigger development. First, the humanitarian sector could scale up the release of prearranged funding based on real-time surveillance data (eg, suspected cases) while other emergency funding is secured. Second, the humanitarian sector could take advantage of weather forecasts and seasonal climate forecasts to anticipate outbreaks linked to extreme climatic events, anomalous climatic conditions or highly suitable climatic conditions. Third, to extend the lead time available for intervention, the humanitarian sector could use observed environmental and socioeconomic transmission risk factors (eg, population displacement, overcrowding, presence of vectors, weather changes) in combination with real-time surveillance data to improve early detection or curb a rapid increase in cases, while other emergency funding is secured. Fourth, data-driven outbreak forecasting using seasonal forecasts can help extend the lead time further to make informed decisions about future risks. We present examples and discuss the trade-offs between approaches. As anticipatory action for outbreaks becomes established, we expect that future applications will integrate all four approaches.

**SUMMARY BOX**

- **‘**Anticipatory action’ in the humanitarian sector involves using early warnings and prearranged funding to act ahead of predicted hazardous events to prevent or mitigate acute humanitarian impacts before they fully unfold.
- The study presents an analytical framework for developing triggers for anticipatory action for climate-sensitive infectious disease outbreaks, informed by practices from organisations such as the Red Cross Red Crescent, United Nations agencies and Médecins Sans Frontières.
- Four trigger approaches are proposed: use of real-time disease surveillance data, use of weather or climate forecasts, contextual risk indicators and data-driven outbreak forecasting.
- Each approach offers trade-offs in terms of lead time and certainty, with the potential for future integration of all four as phased triggers to enhance outbreak preparedness and response as risks evolve.

## Introduction

 Early warning systems systematically offer timely and relevant information to help individuals, governments or organisations take action to reduce their risk of an impending hazard.^1^ Over the past decade, the humanitarian sector has transformed the use of early warnings into a specific ex-ante emergency funding mechanism called ‘anticipatory action’.^2^ Anticipatory action is defined as acting ahead of predicted hazardous events to prevent or mitigate acute humanitarian impacts before they fully unfold.^3^ It is part of a broad strategy to manage risk and is complementary to other preparedness, readiness and response efforts.^4^,^7^ While different actors use distinct names (eg, forecast-based financing, forecast-based action, early warning early action) and financing mechanisms, fundamentally anticipatory action makes taking effective action the default when predicted risks increase, rather than once the humanitarian needs and human suffering have manifested.^6^

Anticipatory action is usually formalised in a plan or protocol which outlines the release of ex-ante emergency funding according to a predefined trigger (of observations or forecast information), in priority intervention areas, to enable preagreed actions that will help mitigate the humanitarian impacts of an impending disaster.^28^,^10^ This formalised plan or protocol clearly defines actor responsibilities to minimise decision-making delays as risks escalate. The plan or protocol is typically active for a defined period (eg, 2–5 years for the Red Cross Red Crescent Movement) and requires periodic updates to remain relevant to evolving risk landscapes.

Since 2014, anticipatory action has mainly been applied to both rapid-onset and slow-onset extreme climatic events, such as tropical cyclones and droughts respectively.^11^ Triggers - the criteria used to release funding and initiate actions - are increasingly developed based on principles of impact-based forecasting, which focuses on the expected impact of weather (eg, flooding affecting 20% of low-lying households) rather than the weather event itself.^4^ For rapid onset events (eg, tropical cyclones, floods), triggers rely on current conditions or forecasts predicting significant damage, with a lead time of 3–10 days. Slow-onset events (eg, droughts) are more challenging to predict; anticipatory actions aim to mitigate severe impacts as the hazard evolves, using phased triggers linked to early warning signals of increasing risk. Over the past decade, anticipatory action has shifted humanitarian systems from reactive to proactive, leveraging reliable climate services.^10^

There is increasing interest in integrating anticipatory action into disease outbreak prevention, preparedness and response to enable humanitarian actors to rapidly scale up interventions as outbreak risk drivers - shaped by interactions between climate, environmental, social and political factors - emerge.^11 12^ Formalising the release of prearranged emergency funding based on early warning signals can help overcome response delays (which can range from days to weeks) improving early detection and curbing rapid increases in cases, while other emergency funding is secured. Acting on early warnings reduces transmission risk, averts cases and upholds the dignity of affected populations. While substantial guidance exists for anticipatory action in rapid-onset or slow-onset climate-related crises, there is a notable gap in guidance on establishing outbreak-specific triggers.

To address this gap, a group of multidisciplinary researchers, including the interagency co-leads of the Anticipatory Action and Health Thematic Working Group hosted at the Anticipation Hub, have collaboratively authored this practice paper. The paper aims to describe and categorise emerging efforts within the humanitarian sector to integrate anticipatory action into disease outbreak preparedness and response. It is based on a comprehensive review of existing and emerging practices from the Red Cross Red Crescent Movement, United Nations (UN) agencies and Médecins Sans Frontières (MSF) since 2014, and the authors’ expert judgement on promising future approaches for developing relevant trigger-based systems tailored to climate-sensitive infectious disease outbreaks.

**Figure 1:**
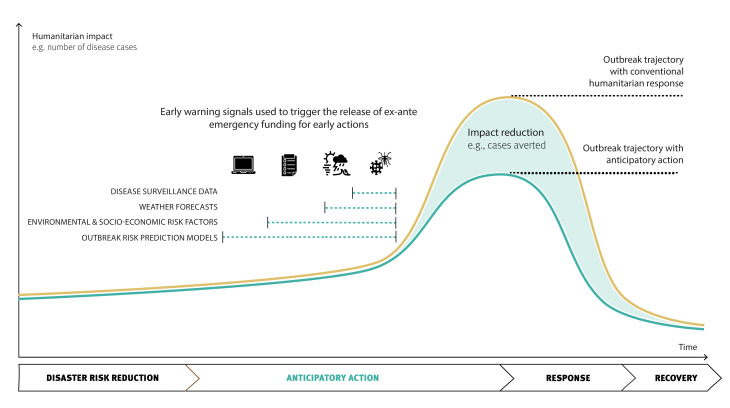
A schematic illustrating the possible triggers for anticipatory action in disease outbreak preparedness and response, and how early action can avert cases and reduce the epidemic curve compared to the trajectory under conventional humanitarian response. Anticipatory action links routine long-term disaster risk reduction strategies with emergency response, as visualised below the x-axis. Note: the timeline is not to scale and has focused on anticipatory action within the disaster risk management continuum; disaster risk reduction typically occurs over years, whereas anticipatory action is triggered in a distinct window of time that may span months, weeks or only days. The lead time available for anticipatory action for climate-sensitive infectious disease outbreaks depends on the target disease and its associated early warning signals used to trigger the release of ex-ante emergency funding for early actions. These early warning signals may include disease surveillance data, weather forecasts, environmental and socioeconomic risk factors, or climate-driven outbreak risk prediction models. Among these, disease surveillance data provides the highest level of certainty regarding escalating outbreak risk. Consequently, it is recommended that surveillance data serve as a fundamental trigger for early action across all trigger-based anticipatory action systems, ensuring a timely and evidence-based response.

Four broad approaches for trigger development are identified: (1) using real-time surveillance data; (2) using forecasts of extreme weather or climatic events; (3) using a combination of observed environmental and socioeconomic transmission risk factors in a phased approach; and (4) using data-driven approaches for outbreak forecasting (figure 1). This practice paper provides illustrative examples of these different trigger-based approaches drawn from real-world cases within and beyond the humanitarian sector. Additionally, it offers guidance on the trade-offs between the different trigger approaches; a decision-tree framework to facilitate practical decisions on which trigger approach is most suitable and feasible in a given context; and potential solutions to address persistent challenges.

### Using real-time surveillance data

Real-time surveillance data underpins outbreak response systems such as the WHO’s Early Warning, Alert and Response System (EWARS).^13^ Once suspected or confirmed cases in a community are identified, these should trigger emergency response, though delays in access to care, data collection or sharing, lack of defined thresholds and funding mobilisation can hinder timely action.^14 15^ For this trigger approach, several different scenarios could be defined depending on the disease of interest. First, a trigger may be based on epidemiological indicators in priority areas, such as five cases of acute watery diarrhoea for suspected cholera or a single confirmed cholera case. Early actions would target specific affected communities with focused measures to break transmission chains. Second, a trigger could be based on epidemiological indicators in bordering regions of neighbouring countries where cross-border movement poses a transmission risk, warranting early actions in the target country. Early actions would target a large area of intervention and should use limited resources for low-regrets or no-regrets actions. Third, a trigger could be designed to respond to the unexpected emergence of known or novel pathogens, including spillover events. Automating the release of prearranged funding based on these triggers and agreed thresholds could reduce response delays, enhance early detection, and interrupt transmission chains, while additional emergency funding is secured.

### Using forecasts of extreme climatic events

Extreme climatic events can result in widespread damage to water and sanitation infrastructure and displace people into overcrowded conditions, increasing the risk of outbreaks where infectious diseases are circulating.^16^,^19^ The humanitarian sector has a long history of responding to the aftermath of extreme climatic events to mitigate disease risk.^20 21^ Yet, it could make greater, intentional use of weather forecasts (such as heavy or low rainfall, storms, high temperatures) and subseasonal-to-seasonal climate forecasts to *anticipate* outbreaks linked to extreme climatic events, anomalous climatic conditions or highly suitable climatic conditions, to support vulnerable populations in reducing their risk of infection.

For example, the Mozambican Red Cross Cyclone Early Action Protocol (the formal plan that outlines the early actions to take when a hazard is expected to impact communities) illustrates how a weather forecast can be used to trigger anticipatory action to prevent or reduce the incidence of diarrhoea and cholera after tropical cyclones.^22^ The Mozambican Red Cross used knowledge of the historical impacts of tropical cyclones on coastal communities to establish the trigger of a Category 3 cyclone with an anticipated speed of 120 km/hour or more at landfall, as indicated by Mozambique’s Insituto Nacional de Meterologica in their 72-hour forecast. This cyclone category corresponds to an event with a return period of 5 years (ie, on average this type of event is expected to occur once in every 5 years) likely to cause significant damage to critical water and sanitation infrastructure. The Mozambican Red Cross has 72 hours lead time before the cyclone makes landfall to implement early actions such as refresher training for community-based volunteers on how to use water purification tablets and other hygiene materials and the distribution of water, sanitation and hygiene (WASH) kits (buckets, mugs, jerrycan, soap, water purification tablets), among other actions, which aim to pre-emptively reduce the risk of waterborne disease transmission in the affected community.

Developing an accurate trigger relies on understanding the link between extreme climatic events and outbreak risk in a specific country or location. A previous review indicates there is strong evidence for an increased risk of cholera and diarrhoeal diseases after floods and tropical cyclones, but mixed evidence for how extreme climatic events influence the risk of vector-borne disease outbreaks.^19^ Sequential climatic events, such as periods of drought followed by heavy rainfall, may be important for certain diseases when developing triggers.^19 23^ Additionally, global climate phenomena such as El Niño Southern Oscillation and specifically the warming El Niño or cooling La Niña phases may be important in influencing extreme climatic events and therefore risk of an outbreak of certain diseases.^24^,^35^ Context-specific research on the interactions between single or sequential extreme climatic events and outbreaks is needed to support the humanitarian sector in making greater use of climate and weather forecasts for anticipatory action for outbreaks. This trigger could also be combined in a phased approach with real-time surveillance data to ensure further targeted early actions are implemented should suspected or confirmed cases emerge in the community to interrupt disease transmission chains.

### Using observations of environmental and socioeconomic risk factors in combination with real-time surveillance data

Using real-time observations of weather and socioeconomic factors in combination with disease surveillance data builds on established WHO EWARS outbreak management practices.^13^ In this trigger approach, several phased triggers or scenarios are defined that integrate observed risk factors for transmission (eg, population displacement, overcrowding, presence of vectors, weather changes) with disease surveillance data.

For example, the anticipatory action framework for cholera in the Democratic Republic of the Congo (DRC), facilitated by UN Office for the Coordination of Humanitarian Affairs (UNOCHA) in partnership with UN Children’s Fund (UNICEF), WHO and the Ministry of Health (MoH), aims to mitigate large cholera outbreaks.^36^ This framework includes three scenarios that can trigger the release of ex-ante emergency funding using real-time observations. The trigger for Scenario 1 is an anomalous number of cholera cases or deaths reported over 3 weeks in any health zone within the endemic provinces of North Kivu, South Kivu, Tanganyika, Haut-Lomami and Haut-Katanga. The trigger for Scenario 2 is an external event (eg, floods or population displacement) that elevates cholera risk in an endemic province. The trigger for Scenario 3 is an anomalous number of cases or deaths reported over 3 weeks in any health zone within a non-endemic province. Epidemiological data is provided by the National Programme for the Elimination of Cholera and the Control of Other Diarrhoeal Diseases. This was the first operationalised anticipatory action system for outbreaks in the humanitarian sector.

In 2023, this framework was activated twice due to anomalous case numbers over 3 weeks in cholera-endemic provinces. The first activation occurred in January in Nyiragongo health zone, North Kivu, and the second in June in Kiambi health zone, Tanganyika.^37^ Early actions by UNICEF, WHO and partners included rapid case detection and confirmation, interruption of cholera transmission through UNICEF’s case area targeted interventions (CATI), promotion of safe hygiene practices and support for cholera case management.^36^

The Red Cross Red Crescent Movement is developing risk matrices that integrate observations of environmental conditions (eg, flooding) with socioeconomic factors (eg, displacement, overcrowding, conflict) and suspected or confirmed cases, using a phased trigger approach. A portion of the ex-ante emergency funding can be used for ‘readiness’ activities once the early action protocol is internally approved, before the triggers are met. These ‘readiness’ activities may include prepositioning of critical supplies, such as chlorination tablets for water treatment. The system, structured around phased triggers, allows the implementing partner (the Red Cross Red Crescent National Society in this case) to predefine specific early actions corresponding to each trigger. For instance, triggers based on environmental or socioeconomic factors, in the absence of suspected cholera cases in the intervention area, may target a broad intervention area and include ‘low-regret’ or ‘no-regret’ actions. These actions, such as hygiene promotion and awareness-raising campaigns, are relatively low-cost yet provide community benefits and help mitigate the potential risk of future outbreaks.

Epidemiological triggers in the intervention area (eg, suspected or confirmed cases, or a certain threshold of, eg, acute watery diarrhoea in the case of cholera) may either follow earlier enviromental or socioeconomic triggers or be independent of prior environmental or socioeconomic conditions. The epidemiological triggers allow for more precise resource allocation to affected communities, enabling targeted efforts to interrupt disease transmission chains. For cholera, early actions activated by epidemiological triggers could include, among others, active case finding, the establishment of oral rehydration points and emergency WASH interventions, such as CATIs. This phased approach ensures that ex-ante funding is flexibly used for a range of early actions tailored to the level of outbreak risk.

### Using data-driven approaches for outbreak forecasting

Data-driven approaches use historical data to identify long-term trends and patterns, to help make informed decisions about present or future conditions. Computational methodologies in these approaches identify lagged correlations between climate variables and disease incidence to forecast outbreak risk.^38^ This means the climatic conditions from the preceding months are used to predict current or future cases.^39^ Forecast timescales can range from short-term (days to weeks) to seasonal (several months).^40^,^44^ The extended time window for early actions provided by long-lead climate-informed outbreak forecasting, as well as the quantified uncertainty, make this a promising trigger approach for anticipatory action.

To date, the humanitarian sector has not operationalised anticipatory action for outbreaks using outbreak forecasting. MSF’s Malaria Anticipation Project, launched in 2021, aims to predict malaria peak timing and intensity in Lankien, Jonglei State, South Sudan via data-driven approaches.^45^ Data analysis and model development using a decade of routine malaria surveillance data (2013–2023) for Lankien, Jonglei State and various meteorological (temperature and precipitation) and environmental variables (vegetation cover) has been carried out.^45^ However, this system is not operationalised, meaning a formalised protocol has yet to be developed for the release of ex-ante emergency funding to enable early action based on a forecast of malaria caseload from the model. Early actions are planned to include a set of preventive measures (vector control, chemoprevention), enhancing clinical capacity, advocating for intervention by other actors and community-led responses.

Developing a trigger for anticipatory action from outbreak forecast models involves defining the ‘outbreak threshold’ and the ‘decision trigger threshold’. The outbreak threshold is context-specific and disease-specific and represents a quantifiable metric (such as the minimum number of cases in a given month and location) to distinguish whether or not an outbreak has occurred.^46^ It is used to assess model performance against historical data. Previous outbreak modelling studies, not in humanitarian crises, have defined outbreak thresholds in a variety of ways, including using: percentiles of disease incidence above historic averages in the same period (75th percentile or 95th percentile); standard deviations of daily disease distribution from the mean; or specific case numbers per population unit, such as 300 cases per 100 000 inhabitants for high-risk.^23 44 47^

The decision trigger threshold determines when the model forecast could be used to activate an intervention. The decision trigger threshold can be set several ways. In some cases, the decision trigger threshold may be the outbreak threshold itself. For example, under International Health Regulations, one confirmed case constitutes an outbreak for diseases such as cholera, yellow fever and viral haemorrhagic fevers.^48 49^ Typically, in the acute phase of a humanitarian emergency, simple outbreak thresholds are used such as 1.5 times a baseline (the baseline may be the average number of cases recorded in the previous 3 weeks) or the emergence of a single case of measles in a camp-like setting.^48 50^ The key model output could be a prediction of the caseload and the decision trigger threshold could be when the model predicts a minimum caseload which surpasses the outbreak threshold.

The decision trigger threshold can also be set through statistical analysis. For example, the decision trigger threshold can be derived from probabilistic outputs of forecasting models, such as those using Bayesian statistical methods, which estimate the probability that an outbreak will occur. The precise value of this decision trigger threshold can be informed by statistical tools like the receiver operating characteristic (ROC) curve, which helps to identify the decision trigger threshold value that serves as the point of maximum distinction between the true positive rate (ie, outbreak occurred and outbreak detected; sensitivity) and the false positive rate (ie, no outbreak occurred but outbreak detected; 1-specificity).

Alternatively, the decision trigger threshold can be set based on expert judgement or guidelines from national health agencies, such as the national MoH. The most appropriate outbreak threshold and decision trigger threshold will have to be co-developed according to the available data, the humanitarian context, existing national or subnational guidance and the resources and response capacity of the organisations involved.

ENDCast (El Niño driven Disease foreCasting) is an early warning framework that integrates seasonal climate forecasts with Bayesian mixed models to produce probabilistic predictions of climate-sensitive infectious disease outbreaks with a 1-month to 6-month outlook.^51^ The tool was designed to rapidly respond to emerging El Niño or La Niña events, or be implemented as part of routine national health planning and response. The initial prototype focused on the Latin American and the Caribbean region during the 2023–2024 El Niño event. This included department-level predictions of dengue outbreaks in Colombia, where the outbreak threshold was defined as the historical 75th percentile of dengue cases per department per calendar month. Outbreak probabilities were categorised into context-specific risk levels—‘Low’, ‘Medium’, ‘High’ and ‘Very High’—reflecting the model’s confidence that an outbreak will occur. These risk levels are designed to correspond with public health interventions mandated by national or regional health agencies to mitigate or prevent epidemic occurrence. In humanitarian anticipatory action, the decision trigger threshold can be set at the outbreak probability that distinguishes risk from ‘Low’ to ‘Medium’ or ‘Medium’ to ‘High’, based on organisational capacity to intervene.

The outbreak risk prediction model could serve as an initial trigger, providing a lead time of several months to weeks to facilitate specific early actions. This could be complemented by a secondary trigger of real-time disease surveillance data, which would enable targeted early actions based on observed epidemiological indicators in prioritised areas.

### Trade-offs between the trigger approaches

Here we proposed four trigger approaches for anticipatory action for outbreaks: (1) using real-time surveillance data; (2) using forecasts of extreme weather or climatic events; (3) using a combination of observed environmental and socioeconomic risk factors in a phased approach; and (4) using data-driven approaches for outbreak forecasting (table 1). Each has distinct advantages and trade-offs. They vary in available lead time for early action, data requirements, analysis complexity and certainty level.

**Table 1:** The four trigger approaches according to specificity of early actions, lead time and certainty level

Trigger approach		Early actions	Lead time	Certainty of outbreak
1	Surveillance data (suspected or confirmed cases) in prioritised areas	Early actions will target specific affected communities with focused measures to break transmission chains.	Days depending on the disease transmission dynamics and incubation period.	High
	Surveillance data from bordering countries with porous borders	Early actions will target a large area of intervention and should use limited resources for low-regrets or no-regrets actions.		
2	Forecast of an extreme climatic event	Specific communities that are likely to be impacted by the extreme climatic event will be targeted with appropriate early actions to mitigate the risk of outbreak of the priority disease.	3–10 days depending on skill of the weather forecasting system/model.	Depends on the link between the extreme climatic event and disease outbreak risk.
3	Phased triggers: Combination of environmental or socioeconomic factors	Early actions will target a large area of intervention and should use limited resources for low-regrets or no-regrets actions.	Potentially weeks, depending on the risk factors of the disease.	Increasing certainty with the use of disease surveillance data.
	Surveillance data (suspected or confirmed cases) in prioritised areas	Early actions will target specific affected communities with focused measures to break transmission chains.		
4	Outbreak risk prediction model	Early actions will target specific hotspot areas at specific periods based on the model predictions; they may include low-regrets or no-regrets actions.	Potentially months to weeks depending on the model formulation.	Depends on the model performance.

Weather forecasts offer a lead time of 3–10 days but come with significant uncertainty of an outbreak occuring, as not all climate anomalies or extreme climatic events result in disease outbreaks, posing a risk of ‘acting in vain’. Combining environmental and socioeconomic observations that conditions are becoming more conducive for disease transmission helps monitor the increased risk of an outbreak, but the occurrence of an outbreak is not guaranteed and this trigger approach poses similar risks of ‘acting in vain’. Real-time epidemiological surveillance provides the highest certainty but with minimal lead time, as the trigger is cases (suspected or confirmed) already present in the population. Its effectiveness depends on robust surveillance systems and access to real-time data (albeit with reporting delays), which may be disrupted during crises. The ability to operationally use epidemilogical surveillance data can also be affected by political sensitivities around suspected cases and political decisions on when to declare an outbreak. Data-driven outbreak forecasting informed by seasonal climate predictions offers the longest lead time (1–6 months) and quantified (un)certainty levels. Yet, models can be prone to errors: either raising a false alarm or missing an event. The establishment of climate-informed outbreak risk models, as outlined by Finch *et al* (2023), demands considerable resources and requires expertise in advanced statistical modelling; moreover, the data-driven approaches are data-hungry and computationally expensive.^39^ Based on our experience, robust modelling approaches require a minimum amount of epidemiological historical data (‘essential data’), as proposed in table 2. The inclusion of additional ‘desirable’ data could strengthen the analysis by helping to account for non-climatic disease drivers, although this might not be essential in all settings.

**Table 2:** Data requirements to develop robust data-driven climate-informed outbreak risk models

Essential data	
Epidemiological	Either anonymised disaggregated (line list) or aggregated (eg, monthly or weekly per administrative unit) notified cases of infectious diseases of interest (eg, dengue, malaria, leptospirosis, cholera or other diarrhoeal diseases). If providing disaggregated (line list), include information on date, location, sex and/or gender, age, occupation, etc (where relevant). If possible, case data should be labelled as suspected or confirmed, with the method of confirmation included. If providing aggregated case counts, indicate the number of cases per administrative unit (eg, national, province, district, municipality) and provide associated shape files or geographical coordinates in the case of point data (village or clinic). If providing location or geo-referenced data, use latitude and longitude (WGS84) or otherwise, indicate which projection system is being used. Historical records should cover the longest time period available, ideally with a minimum of 10 years. If data is spatially resolved and covers an entire country, the time series could be shorter than 10 years.
Hydrometeorological	Daily, weekly or monthly meteorological records from as many (hydro)meteorological stations in the study area as possible for the longest record possible. At a minimum, (hydro)meteorological data should cover at least 1 year before the start date of the epidemiological data to enable the (hydro)meteorological data to be lagged. Where (hydro)meteorological stations are not available, or coverage is limited, remote sensing data or reanalysis models can be used as a substitute or complementary data set. The following variables are typically of interest for climate-sensitive infectious disease modelling: Temperature (mean, minimum, maximum). Precipitation (cumulative, minimum, maximum, intensity). Drought indicators (eg, standardised precipitation index or standardised precipitation and evaporation index). River height (metres above a given reference level). Humidity (relative and/or specific). Wind (speed, direction).
Demographic information	Population data will ideally vary per year and span the same period as the epidemiological data. If census data is available, the closest census before the epidemiological data records began and the closest census to the end of the epidemiological data record could be used. In the case of clinic-level data, indicate the size of the catchment area. Both in terms of size of population and geographical coverage, where available. Indicate the method used to measure or estimate population size and geographical extent.
Desirable data	
Socioeconomic information	Access to sanitation (eg, water supply, refuse collection, toilets). Variables relating to income, housing, education, etc. Accessibility of healthcare, etc
Environmental information	Land cover classification, land use data, soil moisture, topography/altitude, etc. Vegetation health indices. Solar radiation.
Disease control and intervention information	Vector surveillance and vector control data (eg, genetically modified control approaches). Bed net distribution. Vaccination campaigns/coverage. Other intervention measures (indoor residual spraying, mass drug administration, etc).

### Persistent challenges

Low levels of prearranged financing or inefficient physical transfer of prearranged funding, as well as insufficient codevelopment of triggers, lack of agreement on roles and responsibilities, and low data availability, can individually or in combination hinder the integration of anticipatory action for outbreaks.^12^

The existing prearranged funds in the humanitarian sector are relatively low: the Red Cross Red Crescent National Societies can access between US$235 000–590 000 (equivalent to SFr200 000–500 000) from the Disaster Response Emergency Fund (DREF), and the cholera anticipatory action framework in DRC can access up to US$750 000 per scenario from the Central Emergency Response Fund (CERF), members of the START Network (an international network of NGOs) can additionally receive prearranged funding from the START Fund, while MSF has internal prearranged funding.^4 6 36^ Available funds cover only targeted interventions in specific areas, and not nation-wide coverage. Advocacy efforts from the humanitarian health sector are needed to secure and increase the volume of preagreed financing for health crises. Such efforts would help to ensure prompt action is taken in response to escalating risks while other emergency funding is secured, thereby reducing existing delays in response and protecting hard-won development goals.^12 52^

Insufficient codesign and codevelopment between stakeholders can result in overly complex triggers that are difficult to monitor and interpret, or systems that are challenging to implement and maintain.^53^ Though building effective partnerships demands time and effort to create a shared understanding of terms, concepts, pace, ways of working and responsibilities, it is essential for effective anticipatory action systems.^54^ The development of anticipatory action is inherently interdisciplinary, necessitating collaboration among a diverse range of experts. Depending on the specific hazards and impacts that the anticipatory action system aims to mitigate, this collaboration should involve not only disaster risk managers but also climate scientists, social scientists, social protection experts, public health professionals, epidemiologists and finance professionals. Such interdisciplinary efforts are essential to ensure that the trigger-based system effectively reduces delays in response by streamlining decision-making processes based on early warning signs of escalating risks.

Research shows that anticipatory action is most successful when it is nationally driven, nationally owned and integrated with existing early warning systems.^10^ Previous efforts have shown that iterative collaborative workshops, which bring together disaster risk, health, climate practitioners alongside transdisciplinary researchers, play a critical role in fostering stakeholder commitment to the codevelopment of early warning systems. This process helps build trust, ensure credibility and achieve long-term sustainability of climate services for health.^439 54^,^56^

Data limitations present another significant challenge in developing triggers for anticipatory action for outbreaks. In many humanitarian contexts, epidemiological data often lack the necessary historical records, completeness and quality required for predictive outbreak modelling.^57 58^ Consequently, the feasibility of developing outbreak risk prediction models may be limited, necessitating alternative approaches such as trigger-based systems using weather forecasts or real-time epidemiological data from active or passive surveillance systems.

Even when disease data are collected, accessibility by humanitarian actors and research groups may be constrained due to political sensitivities surrounding health data sharing. These complexities present a key distinction between anticipatory action for infectious disease outbreaks and systems designed for hydrometeorological hazards, where data sharing is often less restricted. Since the majority of anticipatory action systems are developed at a subnational level and prioritise high risk areas (eg, the prioritised areas for multisector interventions (PAMI) in the case of cholera risk), practitioners seeking to integrate anticipatory action into outbreak preparedness and response should consider all available data sources, including subnational, local or project-specific data sets that may provide insights into health risks within the targeted areas.

Additional challenges include discrepancies in climate, socioeconomic, demographic and health data reported from multiple sites, reporting delays and the lack of contextual information (eg, changes in case definitions being used). These issues can complicate the interpretation of data and limit the ability to trigger early actions based on real-time or near-real-time case observations.^14^ Overcoming these challenges requires structural changes that extend far beyond the humanitarian system. For example, the absence of consistent historical climate data and weather stations in many regions may limit the feasibility of using weather forecasts as triggers.^59^ In such cases, remote sensing and reanalysis data can serve as alternatives, though additional work may be needed to calibrate and downscale these data sets to areas most relevant for disease transmission and humanitarian intervention. Achieving this will depend on collaboration between transdisciplinary research teams and practitioners in disaster risk, climate and health—both within the humanitarian system and in national government structures.

Limited data and the capacity of implementing agencies to execute early actions within the available lead time represent some of the most significant challenges for anticipatory action in disease outbreaks. In many humanitarian contexts, establishing anticipatory action systems for outbreaks may not yet be feasible. However, some widespread data challenges can be addressed. For instance, data sharing remains a persistent issue; currently, only 14% of WHO Member States have formal agreements in place for data sharing between national hydrometeorological services and health ministries,^60^ which hinders efforts to develop effective triggers. Concerted and sustained efforts to bring these actors together, with a focus on improving data sharing to enhance climate-sensitive health outcomes, are urgently needed. The humanitarian sector can play a pivotal role in advocating for such initiatives, encouraging WHO Member States to adopt and implement these agreements, emphasising their potential to reduce predictable and preventable climate-related humanitarian crises, including climate-sensitive infectious disease outbreaks and alleviate human suffering.

Ongoing efforts to develop open-source digital tools to harmonise health, climate, environmental and socioeconomic data offer promising solutions to data integration challenges.^61^,^63^ However, more investment is needed to strengthen disease surveillance systems and expand weather stations coverage in under-resourced settings, targeting hotspot areas for disease risk, social vulnerability and hazard exposure. Additionally, capacity strengthening of regional and local actors is needed to enable them to effectively use climate services for health and independently analyse their existing data using open-source tools. Equally important is community engagement and empowerment, which can validate data and ensure its relevance to local contexts. This is particularly valuable in settings where political sensitivities may delay the official declaration of disease outbreaks. Epidemiological triggers should be grounded in local realities rather than relying solely on official outbreak declarations, which may come too late and necessitate full emergency response funding.

**Figure 2:**
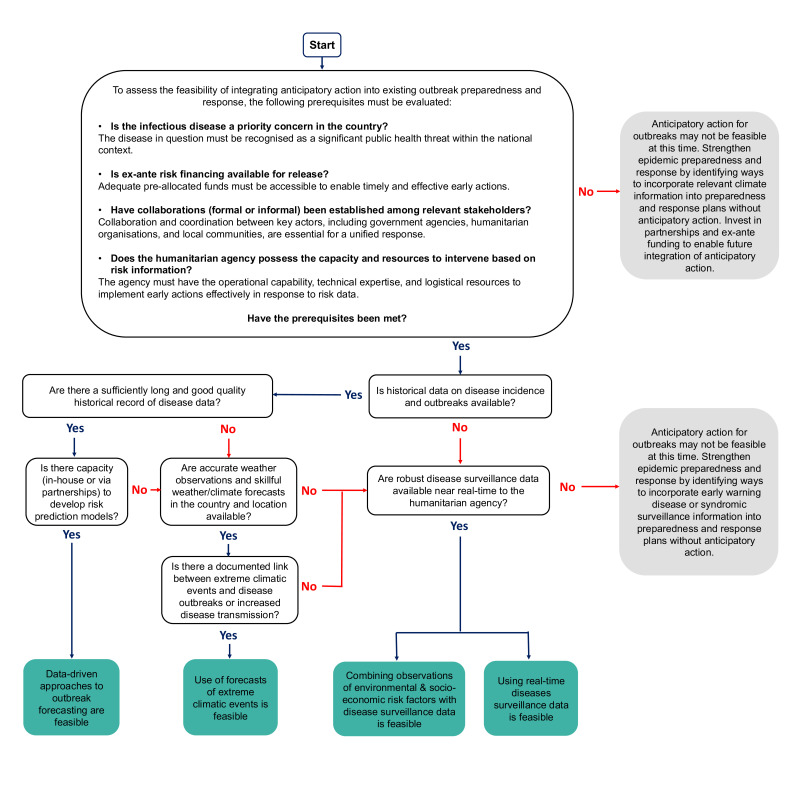
Decision tree to support the choice and development of the most appropriate trigger for anticipatory action for an outbreak. Historical data can include number of historical outbreaks, caseload and number of deaths during previous outbreaks, monthly time series of disease data, drivers of disease transmission. Surveillance data could include community-based surveillance, regional/district-level surveillance data from MoH, Public Health Institute (PHI), WHO/Pan American Health Organisation (PAHO) or other non-governmental organisations (NGOs). The link between extreme climatic events and infectious disease outbreaks varies by country and disease. At a minimum, there is strong evidence from outbreak investigations, academic papers and past reports that certain climatic events have previously triggered outbreaks.

Ultimately, the feasibility of developing an effective trigger mechanism for anticipatory action is influenced by institutional capacities, stakeholder interests and, importantly, data availability. We propose a decision tree to identify the most suitable trigger according to data availability and capacity (figure 2). If neither a sufficient time series of good quality disease and climate data nor reliable surveillance data is available, a trigger based on extreme climatic events may be most appropriate. Conversely, if historical data are lacking but reliable real-time surveillance exists, combining surveillance data with environmental and socioeconomic risk factors may be feasible. When data availability is high and analytical capacity is sufficient, developing a data-driven outbreak forecast model becomes viable.

Finally, although beyond the scope of this practice paper—which focuses on providing guidance for developing triggers—the selection of early actions and the systematic evaluation of their impact will be critical for future learning and improvement in anticipatory action for disease outbreaks.

## Conclusion

The integration of anticipatory action into outbreak preparedness and response holds substantial potential to improve the timing of interventions and reduce delays in response. Unlike anticipatory action for extreme climatic events, which mitigate but do not prevent the event, anticipatory action for outbreaks has the potential to contain disease spread early, and, in exceptional cases, to prevent the outbreak from occurring in the first place. Integrating anticipatory action into outbreak preparedness and response carries numerous policy implications. These include encouraging WHO Member States to move beyond the current 14% adoption rate by establishing formal data-sharing agreements between national hydrometeorological services and health ministries. Additionally, investments are needed to strengthen disease surveillance systems, expand the deployment and maintenance of weather stations and address structural barriers to data availability. Strengthening the capacity of national actors to form transdisciplinary anticipatory action task forces is also critical to develop and use climate services for health, ultimately reducing the risk of humanitarian health crises. Furthermore, dedicated funding mechanisms for anticipatory action must be established to ensure resources are readily available when triggers are activated. This would prevent delays in response during the critical early stages, when it is still possible to contain an outbreak and prevent it from escalating into a large-scale epidemic. Anticipatory action enhances outbreak preparedness and strengthens health system responsiveness, with potential applications in the WHO Pandemic Preparedness Treaty. While categorising four trigger approaches is conceptually helpful, we expect that as anticipatory action for outbreaks becomes more established into routine outbreak preparedness and response planning, it will integrate multiple trigger approaches as appropriate. In many humanitarian settings where data is scarce or unreliable, the development of anticipatory action triggers for outbreaks may not be feasible. However, a shift in mindset from traditional reactive responses to proactive use of available risk information is imperative to reduce preventable suffering.

## Data Availability

Data sharing not applicable as no datasets generated and/or analysed for this study.
